# Advances in 2D Molybdenum Disulfide Transistors for Flexible and Wearable Electronics

**DOI:** 10.3390/mi15121476

**Published:** 2024-12-05

**Authors:** Kyoungwon Kwak, Hyewon Yoon, Seongin Hong, Byung Ha Kang

**Affiliations:** 1 Department of Semiconductor Engineering, Gachon University, Seongnam 13120, Republic of Korea; 2 Department of Physics, Gachon University, Seongnam 13120, Republic of Korea; 3 Department of Chemical Engineering, Massachusetts Institute of Technology, Cambridge, MA 02139, USA

**Keywords:** flexible electronics, wearable electronics, transistors, 2D materials, transition metal dichalcogenides (TMDs), molybdenum disulfide (MoS_2_)

## Abstract

As the trajectory of developing advanced electronics is shifting towards wearable electronics, various methods for implementing flexible and bendable devices capable of conforming to curvilinear surfaces have been widely investigated. In particular, achieving high-performance and stable flexible transistors remains a significant technical challenge, as transistors are fundamental components of electronics, playing a key role in overall performance. Among the wide range of candidates for flexible transistors, two-dimensional (2D) molybdenum disulfide (MoS_2_)-based transistors have emerged as potential solutions to address these challenges. Unlike other 2D materials, the 2D MoS_2_ offers numerous advantages, such as high carrier mobility, a tunable bandgap, superior mechanical strength, and exceptional chemical stability. This review emphasizes the novel techniques of the fabrication process, structure, and material to achieve flexible MoS_2_ transistor-based applications. Furthermore, the distinctive feature of this review is its focus on studies published in high-impact journals over the past decade, emphasizing their methods for developing MoS_2_ transistors into various applications. Finally, the review addresses technical challenges and provides an outlook for flexible and wearable MoS_2_ transistors.

## 1. Introduction

Transition metal dichalcogenides (TMDs) were initially identified in the 1960s, with molybdenum disulfide (MoS_2_) layers being the first ultrathin TMD investigated [1,2,3,4]. Subsequently, the focus on two-dimensional (2D) TMDs expanded after a method of mechanically exfoliating graphene to an atomic thickness was reported in 2004 [5]. Although 2D graphene exhibits exceptionally high electron mobility and low resistance, there are challenges in adjusting the electrical characteristics and applying them to various electronic devices because of the absence of a band gap. Therefore, researchers have started to explore other 2D materials, including TMDs with the same methods, owing to their high performance and potential for various applications.

In the early 2010s, the MoS_2_ monolayer was investigated and applied to transistors. Subsequent research focused on the electrical, optical, and mechanical properties to understand its behavior at the 2D level [6,7,8,9,10,11,12]. Unlike graphene, 2D MoS_2_ possesses a band gap of 1.8 eV, with the band gap energy increasing as the layer becomes thinner. In addition, the single-layer MoS_2_ features a direct bandgap, offering solutions to the challenges faced by conventional Si semiconductors, including heat generation and low efficiency for use as optoelectronics. Moreover, the layered structure of 2D MoS_2_ consists of separate layers held together by van der Waals forces in layer-by-layer stacking. Its structural properties enable mechanical exfoliation and allow it to be extensively employed as channel material without a doping process. The 2D MoS_2_ transistor exhibited silicon-level electrical properties, including a high on/off ratio of approximately 10^8^ and carrier mobility around 500 cm^2^ V^−1^ s^−1^, along with remarkable optical properties and flexibility [10,13,14,15].

Furthermore, 2D MoS_2_ has an outstanding electric field effect while maintaining flexibility. The 2D MoS_2_ transistors were conventionally fabricated using mechanical exfoliation and chemical vapor deposition (CVD). Through the fabrication method, 2D MoS_2_ transistors were constructed on a variety of substrates, ranging from rigid to polymer material. In contrast to the rigid silicon, polymer-based 2D MoS_2_ transistors exhibited excellent bending radius (5–12 mm) and strain (<2%), which indicates their high potential for flexible and wearable electronics [16,17,18]. Consequently, extensive studies have applied 2D MoS_2_ to various electronics, such as sensors, optoelectronics, memory devices, and flexible devices, surpassing the mere implementation in transistors [19,20,21,22,23,24,25,26,27]. In recent years, research on 2D TMDs, including MoS_2_, has focused on enhancing the large-area scalability, stability, and efficiency of practical devices. Moreover, researchers are actively exploring the application potential of 2D TMDs and working on their integration into advanced applications such as flexible electronics, wearable sensors, quantum computing, and energy storage.

As the era demands advanced technology, the trajectory of electronic device development is shifting toward wearable devices [28]. The rising demand for flexible and wearable electronics stems from the necessity to apply devices that can conform to the human body contours, enabling applications in health monitoring, fitness tracking, communication, and beyond. Similarly, the expectations of customers extend to implantable devices, such as lenses equipped with integrated sensors in the medical field, foldable mobile phones, rollable TVs, and screens that can be worn directly on the body in the display field. Research on suitable flexible substrates to improve the performance of flexible devices is increasing rapidly. Polyethylene terephthalate (PET), polyether sulfone (PES), polyimide (PI), and polydimethylsiloxane (PDMS) have been highlighted as flexible substrate materials. Among them, PI substrates with excellent heat resistance and insulation properties are used widely [29]. On the other hand, in the advancement of flexible and wearable electronics, it is essential for substrates and components like transistors, dielectric layers, and interconnects to possess flexibility. Specifically, achieving flexibility in a transistor, the fundamental unit device of electronics, is challenging because of the difficulty in making all components of the delicate transistor (channel, gate insulator (GI), and electrodes) flexible. Indeed, the electrical performance of flexible electronics depends on the materials of transistors, so extensive research on flexible semiconducting material for channels has been conducted to achieve high performance with high flexibility. Among them, 2D TMDs have high flexibility, exhibiting a Young’s modulus of ~200 GPa because of the covalently bonded in-layer atoms and the layer attached by van der Waals forces. [30,31]. This study focused on flexible electronics using 2D MoS_2_ transistors because 2D MoS_2_ was discovered first and has been studied extensively for its outstanding performance among 2D TMD materials.

This review summarizes recent advances in the flexible and wearable MoS_2_-based transistors and their application electronics with the technical concepts, fabrication methods, and electrical characteristics of the representative studies. The distinguishing point of this review is that this study focused on studies published less than ten years ago in high-impact journals and their applications. Section 2 introduces flexible MoS_2_-based transistors, which are the basic unit of electronic devices. The techniques using MoS_2_ are classified and explained according to the application, such as sensors, displays, memory devices, logic circuits, and neuromorphic devices. The contents focused mainly on the detailed materials (e.g., substrates, electrodes, and insulators), core processes, and structures that enable flexible and wearable properties and showed the performance of the devices to realize each target application. Section 3 summarizes the advantages and limitations of the MoS_2_ material for flexible and wearable electronics. The representative types and the status of recent wearable devices using MoS_2_ are also mentioned. Section 4 reports the current technical issues and suggests future research directions for further improvement on the flexible and wearable TMD-based electronics, including MoS_2_.

## 2. Discussion

**Figure 1:**
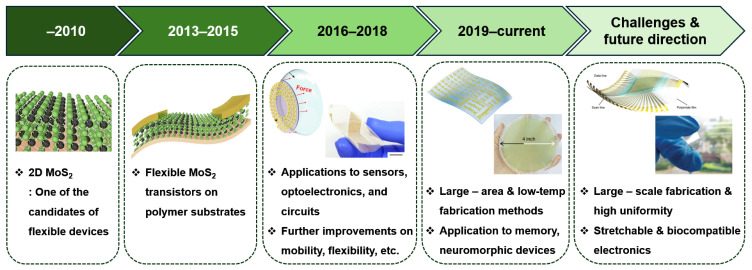
Schematic of recent advances in flexible MoS_2_ electronics [2,18,32,33,34,35].

Figure 1 summarizes the recent advances in flexible MoS_2_ electronics. Before 2010, MoS_2_ was identified as a promising material for flexible electronics [2]. From 2013–2015, transistors on polymer substrates were developed, enabling more complex applications [18]. Between 2016–2018, applications expanded to sensor and optoelectronics, with enhanced flexibility and mobility, etc. [32,33]. Since 2019, large–area fabrication and applications such as memory and neuromorphic devices, have been achieved [34]. Looking forward, future goals will focus on large–scale fabrication with high uniformity [33,35].

### 2.1. Transistors

**Figure 2:**
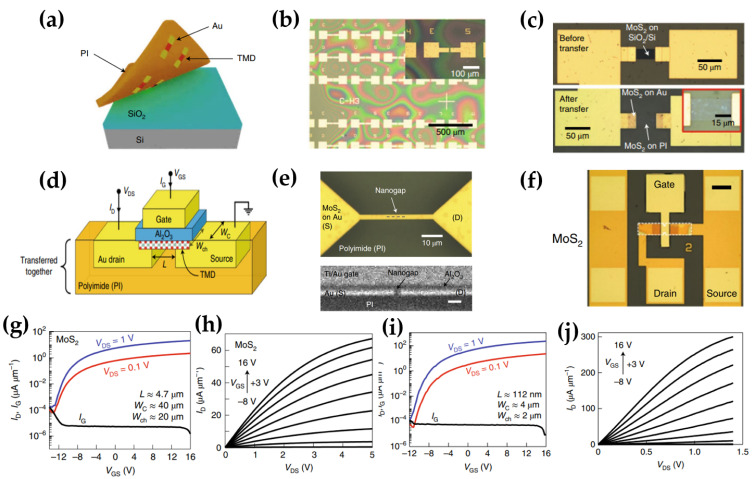
Flexible MoS_2_ FETs. (**a**) Graphic of the transfer process, showing the release of the PI substrate from the SiO_2_/Si substrate. Optical microscopy images of (**b**) contacts on the SiO_2_/Si substrate, (**c**) top-view images of MoS_2_ FETs before and after transfer. (**d**) Schematic diagram of transferred top-gate MoS_2_ FET. (**e**) Nanoscale channel visualization in optical microscopy (top view) and cross-section scanning electron microscopy photograph of the transistor. (**f**) Microscale MoS_2_ FET (type B) optical microscopy images, (**g**) transfer curves, and (**h**) output curves of microscale MoS_2_ FETs (type B). (**i**) I_d_-V_d_ curves and (**j**) I_d_-V_d_ curves of nanoscale MoS_2_ FETs (type B) [49].

The transistor is the fundamental unit device of various electronics, and methods for realizing flexible transistors using TMDs have been widely investigated [18,36,37,38,39,40,41,42,43,44,45,46,47,48]. Daus et al. proposed a flexible nanoscale transistor using TMDs for high-functional flexible electronics [49]. The flexible monolayer MoS_2_ transistors show on-state currents with a maximum of ~470 μA μm^−1^ at V_d_ = 1 V. The transistor is composed of transferring the MoS_2_ and metal contacts onto a PI substrate. After TMD growth using CVD on SiO_2_/Si substrate, Au metal contact was patterned on top by lithography, and PI was then spin-coated on the pre-patterned structures. The device was agitated in DI water, and the PI substrate was released with TMDs and Au from the rigid substrate, as shown in Figure 2a. Such fabrication approaches are suitable for MoS_2_, WSe_2_, and MoSe_2_ WSe_2_ field-effect transistors (FETs). Only patterning contacts before transfer reduces the number of process steps on unprotected TMDs, but results in larger channel widths compared to the electrode widths, as shown in Figure 2b, which is referred to as type A. Type B denotes the approach in which the channel is pre-patterned with reactive ion etching (RIE) before the transfer process, ensuring precise of the channel width, as illustrated in Figure 2c. An aluminum oxide (Al_2_O_3_) gate dielectric is then deposited before the next patterning steps. After gate metal deposition, the device has a top-gate staggered geometry in Figure 2d. For Type A devices, RIE is used to pattern both the channel and gate dielectric after gate metal deposition, whereas Type B devices undergo RIE patterning before the transfer. The cross-sectional view of the nanoscale device (Figure 2e) reveals that the Al_2_O_3_ gate dielectric effectively covers a ~100 nm nanogap and source-drain electrodes. Micrometer scale MoS_2_ FET in Figure 2f was not required for rectification because it optimizes the geometry and modifies the manufacturing process. The MoS_2_ FET better quality allowed a larger drain current (I_d_) = 67.3 µA µm^−1^ in a ~4.7-μm-long FET at V_d_ = 5 V, as shown in Figure 2g,h. The long channel (~112 nm) type B device showed an I_on_/I_off_ ratio of >10^6^, I_d_ ≈ 303 µA µm^−1^ at a drain voltage (V_d_) of 1.4 V, and an extrinsic field-effect mobility (µ_FE_,_ext_) of approximately 8.1 cm^2^ V^−1^ s^−1^, as shown in Figure 2i,j. Furthermore, the MoS_2_ FET showed high performance on flexible substrates, achieved through a transfer process utilizing nano-patterned contacts. This method enabled a device channel as short as ~60 nm, and the device indicated drive currents ~470 μA μm^−1^ at V_d_ = 1 V.

**Figure 3:**
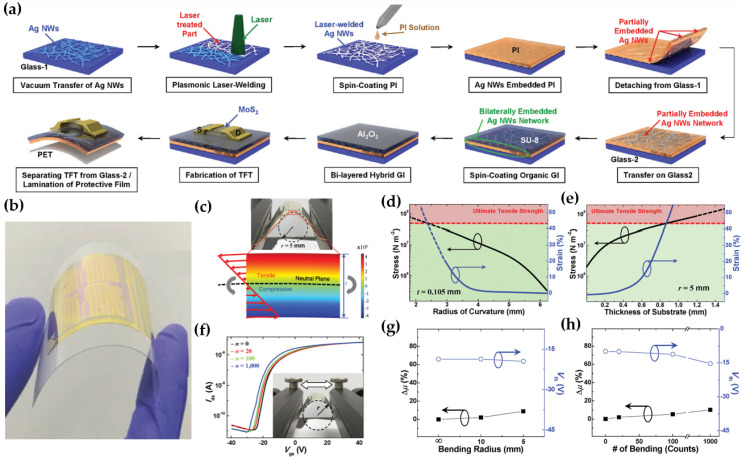
(**a**) Manufacturing a schematic diagram of the flexible MoS_2_ TFT with a laser-irradiated Ag NW network and hybrid GI. (**b**) The Photograph of MoS_2_ TFT on PI substrate. (**c**) The hyper-elastic nonlinear stress analysis is based on experimental data under a 5 mm bending radius. Expected mechanically stable ranges (green area) regarding (**d**) the radius of curvature (**e**) and thickness based on the Mooney–Rivlin model to determine the practical design limits. (**f**) Transfer curves with respect to the number of bending cycles. The variations of µ_eff_ and V_th_ depend on the (**g**) static bending radius and (**h**) the number of bending cycles [50].

Song et al. proposed flexible multilayer MoS_2_ transistors fabricated on solution-processed PI substrates [50]. This study focused on the methods for implementing flexible gate electrodes, gate insulators, and the adoption of a neutral plane. Ag NWs, which act as a gate electrode, were formed by laser welding, as illustrated in Figure 3a. The laser-treated Ag NW network had an electrical sheet resistance that was 37 times lower than the resistance of Ag NW networks without laser treatment. The PI solution was spin-coated on the Ag NW network and penetrated between the nanowires. Subsequently, a PI was firmly cured and peeled off from the glass. A hybrid GI layer (SU-8/Al_2_O_3_) was then deposited on the Ag NWs embedded PI. The thin-film transistor (TFT) array was fabricated by transferring multilayered MoS_2_ flakes, mechanically exfoliated, into the Al_2_O_3_ film and depositing electrodes. Interestingly, 100 µm-thick PET film, a supportive layer, was laminated under the substrate of flexible MoS_2_ TFT, which enables the TFTs to lie within the neutral plane where the material undergoes minimal tensile and compressive stress, as shown in Figure 3b,c. To prevent structural destruction, stress should be in the green area, and the thickness or bending radius of the substrate should be adjusted, as shown in Figure 3d,e. Therefore, the I-V characteristics of the MoS_2_ TFT remained largely consistent compared to those under flat conditions in Figure 3f. The curve shifted slightly as the number of bends increased, but the I_on_/I_off_ ratio maintained a constant of ~10^5^. The variation of µ_FE_ and threshold voltage (V_th_) value depending on the flat state to the bending state (r = 5 mm) was +8.8% and −0.8 V, respectively, as shown in Figure 3g. With the bending cycles of 20, 100, and 1000 under the bending radius of 10 mm, the variation of µ_FE_ increased by 1.9%, 5.3%, and 10%, as the V_th_ was shifted to −10.1 V, −11.3 V, and −15.4 V, respectively, as shown in Figure 3h.

### 2.2. Sensors

**Figure 4:**
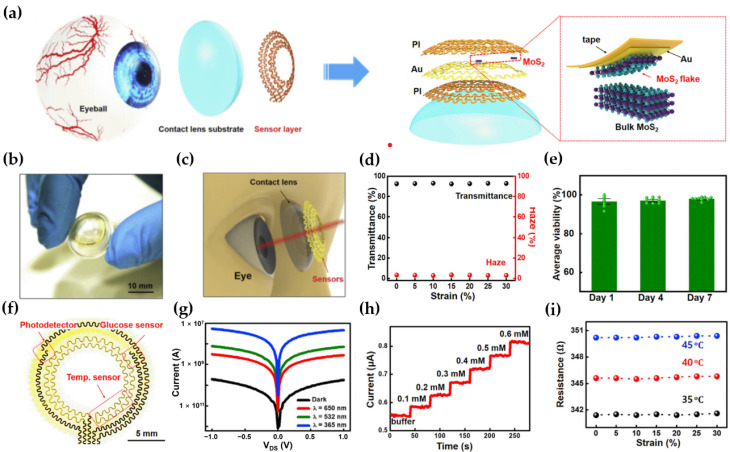
Contact lenses are based on a flexible MoS_2_ transistor with various functions. (**a**) The schematic diagram of each layer of contact lenses. The dashed region shows the mechanically exfoliated flakes of MoS_2_. (**b**) Photograph of a flexible MoS_2_ sensing layer on a dome-shaped PDMS substrate. (**c**) Schematic diagram of a smart contact lens and its dimensions correspond to the human eye. (**d**) Optical characteristics of the lenses at strain from 0 to 30%. (**e**) Cytotoxicity tests determining biocompatibility. (**f**) Structure of the system composed of various sensors with winding mesh structures. (**g**) Photocurrent curves with respect to wavelengths. (**h**) Current variation of the glucose sensor with respect to the glucose concentration. (**i**) Resistance variation of the temperature sensor with respect to the strain condition [32].

2D TMDs have found extensive application in various sensor devices due to their remarkable selectivity and sensitivity [51,52,53,54,55,56,57,58,59,60,61,62,63,64]. Guo et al. proposed flexible contact lenses with various sensor functions using a winding mesh system and ultra-thin MoS_2_ transistors [32]. To optimize the contact lens device configuration, the fabrication of back-gate MoS_2_ FETs was performed on SiO_2_/Si substrates and their performance was evaluated. The MoS_2_ FETs demonstrated a high I_on_/I_off_ ratio exceeding 10^7^ at V_d_ = 5 V, confirming their suitability as contact lens FETs. As shown in Figure 4a, a PI layer was spin-coated onto the glass substrate to construct contact lens, and MoS_2_ flakes were transferred using a gold-mediated exfoliation method. Subsequently, Cr (5 nm) and Au (150 nm) layers were deposited, patterned, and coated with another PI layer to serve as a passivation layer. Finally, the layer of sensor was suspended in DI water and then transferred to the PDMS layer. In addition, the whole lens was heated in a vacuum chamber to eliminate moisture and air bubbles, ensuring a firm bond between the sensor layer and the underlying substrate. Figure 4b shows that the sensor, integrated on a 30 μm PDMS substrate, exhibited strong bonding and adhesion to the surface. Figure 4c further highlights that the sensor does not obstruct the pupil, ensuring the electrodes do not interfere with the field of view. Also, the deformation of the sensor area was 3.0%, and the deformation of the MoS_2_ area was less than 0.5%, even though the maximum strain was applied. For the transparency of the lenses, optical transmittance was measured under tensile strain conditions of up to 30%, and the transmittance of over 90% was then maintained over the entire range in Figure 4d. The lenses were tested for cytotoxicity, and more than 95% of the cells survived in the seven-day analysis. Hence, the lenses are harmless to the human body in Figure 4e. Each sensor (MoS_2_ photodetector, glucose sensor, and temperature sensor) was interconnected with Au electrodes and fabricated as a mesh system around the periphery to prevent hindering the visual field. The mesh structure provided excellent elasticity and kept the plane neutral, allowing the uniaxial strain to approach zero as the structure deforms in Figure 4f. The contact lenses capable of multimodal sensing typically have three functions (photodetector, glucose detector, and temperature sensor), each of which operates on a different mechanism of FET coupling. Like the photodetection mechanism of conventional MoS_2_ transistors, when bias and light are applied to the Au electrode and MoS_2_ channel with a Schottky barrier, electrons gain energy and transition to the conduction band, leading to an increase in current flow. The I–V characteristics were measured for each wavelength to monitor the wavelengths harmful to the eyes. The photocurrent (I_ph_) at a wavelength of 650 nm was smaller than the I_ph_ at 365 nm and 532 nm, meaning that a shorter wavelength results in a higher transition probability, as shown in Figure 4g. For the glucose sensor function, glucose oxidase (GOD), a glucose-sensing bioenzyme, was immobilized on the MoS_2_ surface to enable glucose recognition and enhanced conductivity when in contact with tear fluid. When glucose in the tear fluid is oxidized by GOD, H_2_O_2_ is formed, which reacts with oxygen to produce electrons (e^−^) and hydrogen ions (H^+^). The generation of free electrons from this reaction leads to an increase in the conductivity of the MoS_2_ FET, and this current change is proportional to the glucose level. The sensitivity was measured within the range of glucose concentrations found in tears. The glucose meter showed high sensitivity and stability to concentration, as shown in Figure 4h. Finally, the temperature sensor, made from thin Au wires, measures the eye surface temperature and demonstrates the high sensitivity of 0.94 Ω/°C. In Figure 4i, the strain of the sensor was almost unchanged at ocular surface temperature.

**Figure 5:**
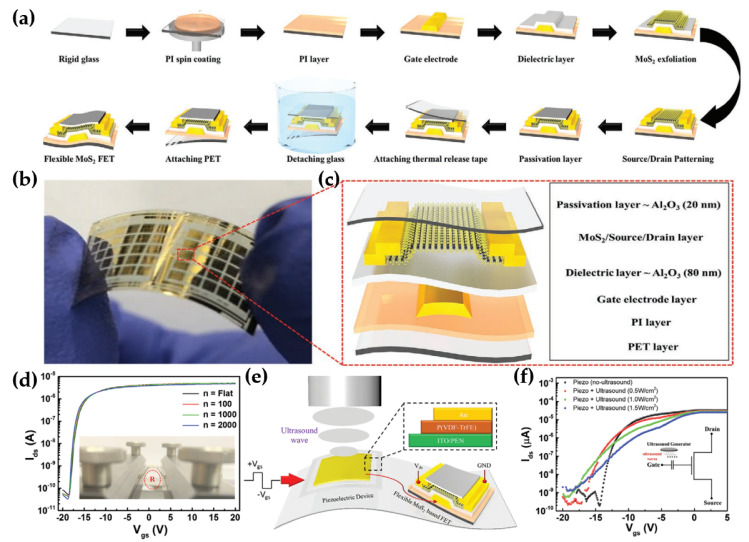
Flexible MoS_2_ FET of ultrasonic detector. (**a**) Fabrication step of flexible MoS_2_ FET. (**b**) Photograph of the device. (**c**) Schematic layout of the device. (**d**) I-V curves of MoS_2_ FET for repetitive bending stress. (**e**) Schematic diagram of the ultrasound detection system. (**f**) Transfer curves of ultrasonic detectors with a piezoelectric device [65].

Wearable electronics require high-performance ultrasonic detectors with flexibility. Naqi et al. proposed a flexible MoS_2_ FET for ultrasonic detectors, featuring an inverted-staggered bottom-gate transistor design on a PI substrate to address existing limitations [65]. To fabricate flexible MoS_2_ FET, a PI solution was spin-coated onto the rigid glass substrate, followed by gate terminal patterning using photolithography. Next, an 80 nm-thick Al_2_O_3_ dielectric layer was deposited on that gate electrode with ALD, and the MoS_2_ channel was exfoliated. Then, electrodes were patterned using a lift-off process, and an Al_2_O_3_ layer (20 nm) was deposited as a passivation layer. Initially, the rigid glass substrate supported the MoS_2_ FET fabrication before transferring it to a flexible substrate. Thermal release tape was attached to the device and immersed in DI water set to 70 °C to separate the glass substrate. PET was attached to the PI substrate, and the attached thermal release tape was placed on a hot plate heated to 90 °C. A completely flexible MoS_2_ FET was obtained through the manufacturing process as shown in Figure 5a–c. In Figure 5d, the flexible MoS_2_ FET characteristics barely changed under 2000 bending cycles at a radius of 5 mm, demonstrating the high flexibility of the device. The flexible MoS_2_ FET, an extended gate approach, was used in ultrasound detection by linking it to a self-assembled piezoelectric device composed of P (VDF-TrFE). The connection of the piezoelectric detector to the flexible FET is pivotal for achieving ultrasound input in FET output characteristics, as shown in Figure 5e. Ultrasound stimulus waves with frequencies of 10, 100, and 500 kHz at a power density of 1.5 Wcm^−2^ were applied to the piezoelectric layer, which induced the gradual shift of V_th_ and a decrease in the maximum I_d_ (Figure 5f). Similarly, the device slightly decreased in I_d_ as the ultrasound frequency increased across the tested ranges (10, 100, and 500 kHz). These results highlight the potential of this system as a novel platform for advancing rapid ultrasonic sensing, photoacoustic imaging, medical imaging technologies, and object identification systems.

**Figure 6:**
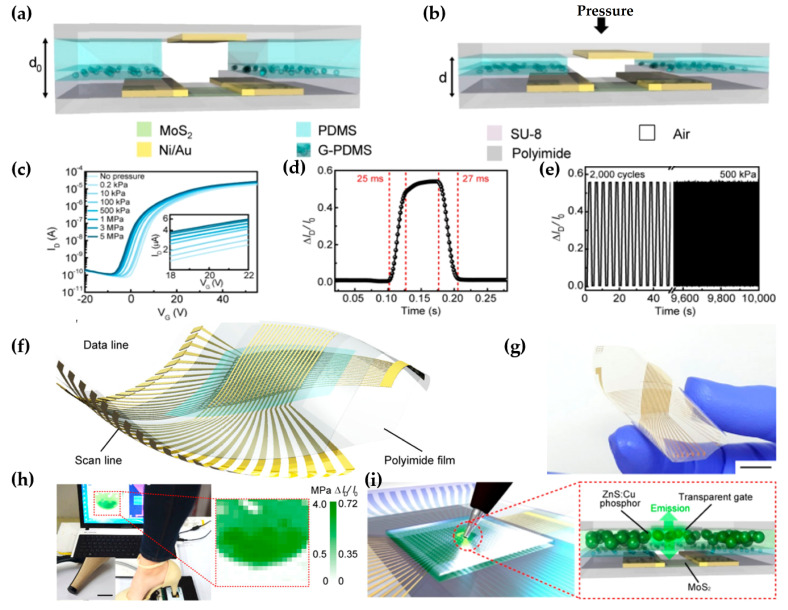
Air-dielectric MoS_2_ FET of the active-matrix pressure sensors. (**a**) Cross-section of the MoS_2_ FET pressure sensor. (**b**) Side view schematic of the device under pressure. (**c**) Series of transfer curves (V_d_ = 1 V, V_g_ = 20 V) of MoS_2_ FET according to a change in pressure. (**d**) Response and recovery time. (**e**) Reliability test. (**f**) Schematic image and (**g**) photograph of the flexible active-matrix array. (**h**) Footstep pressure measurements using a shoe heel. (**i**) Schematic images of the pressure sensor with phosphor ZnS:Cu particles [33].

Jang et al. proposed the flexible active-matrix pressure sensors comprised of mechanoluminescent and air-dielectric MoS_2_ FETs to detect a broad spectrum of pressures [33]. Pressure-sensitive MoS_2_ FET, the unit device of an active-matrix array, was composed of tactile pressure sensors using luminescent particles and an air dielectric. The pressure detection range of the pressure sensor was broadened by attaching a flat PDMS film to the planar G-PDMS layer, uniformly dispersing glycerol microdroplets within the PDMS layer, and forming a double-layer elastomer spacer, as illustrated in Figure 6a,b. The transfer curves in Figure 6c showed an increase in I_d_ and negative shifts of V_th_ when higher pressure was applied. In Figure 6d, the loading-unloading test indicated response and recovery times of 25 ms and 27 ms, respectively. Furthermore, the pressure sensor exhibited stable performance even after 2000 cycles of periodic pressure loading at 500 kPa (Figure 6e). The detection of a target spatiotemporally was solved by integrating the air-dielectric MoS_2_ FET and Cu-doped ZnS (ZnS:Cu) phosphor particles to form a flexible active-matrix array, as shown in Figure 6f,g. A 20 × 20 active-matrix array facilitated real-time measurement and spatial mapping of pressure distribution exerted by the heel footstep. The distribution of footsteps was visualized using a color gradient contour plot (Figure 6h). The visible light emitted by Mechanoluminescence (ML) materials could generate extra photocurrents to the air dielectric MoS_2_ FET, further increasing the pressure sensitivity. ZnS:Cu phosphor particles were synthesized and dispersed into PDMS to make the internal light emissions uniform. The pressure on the elastomer layer was transferred to the Zn crystal to induce light emission. Therefore, the light illuminated the MoS_2_ channel, and the loaded pressure could be observed visually in Figure 6i.

**Figure 7:**
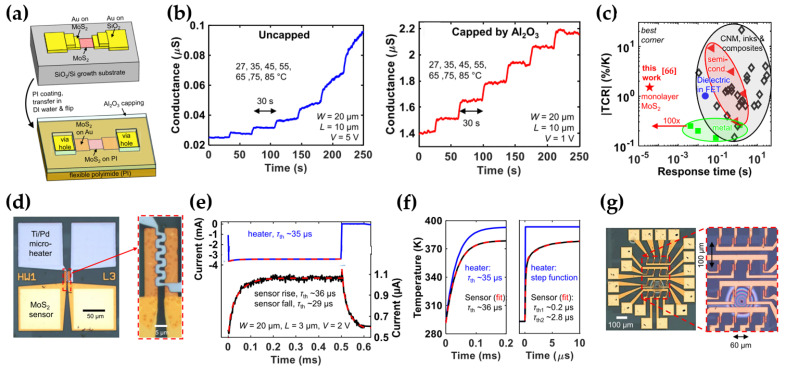
Rapid-response flexible MoS_2_ temperature sensors with a micro heater. (**a**) Schematic diagram of fabrication and structure of the MoS_2_ sensors. The color of MoS_2_ was expressed differently depending on the thickness of Au. (**b**) Comparison of the performance before and after Al_2_O_3_ encapsulation of the sensor. (**c**) Comparison of the thermal coefficient of resistance (TCR)-response time performance with other flexible temperature sensors. (**d**) The overall structure of the microheater embedded in the temperature sensor. The dotted line on the right is the active area of the sensor and heater. (**e**) Response characteristics when pulsed with a heater. (**f**) The simulated response of a heater with τ_th_ = 35 μs on the left and the step function on the right. (**g**) Optical image of the 4 × 4 temperature sensor array [66].

Daus et al. proposed the fast-response flexible monolayer MoS_2_ temperature sensors [66]. Although temperature sensors on flexible substrates have been proposed in various fields, the actual development of sensors with a rapid response of milliseconds remains a challenge because of the thermal mass of encapsulation in sensors. Daus et al. fabricated a temperature sensor that compensates for this disadvantage using a monolayer MoS_2_ and Al_2_O_3_ encapsulation. MoS_2_ films were patterned by depositing Au metal on a spin-coated flexible PI substrate (Figure 7a). The Al_2_O_3_ encapsulation layer and Ti/Pd were deposited sequentially, which acted as a gate dielectric layer and gate electrode, respectively. The conductance of the uncapped MoS_2_ temperature sensor was not saturated but continued to increase after the temperature reached the operating point. On the other hand, the conductance of the Al_2_O_3_-capped sensor showed stable operation, perfectly following the programmed temperature in Figure 7b.

The conductance increased ∼50-fold after Al_2_O_3_ encapsulation due to the reduction of surface exposure to oxygen and water, as well as the n-type doping effects induced by Al_2_O_3_ [67,68]. Temperature sensors based on ink, carbon nanomaterials (CNMs), and composites have a large temperature coefficient of resistance (TCR) (Figure 7c), whereas they showed a slow response time, typically ranging from 0.1 to 10 s. The metal thin-film sensor group showed the fastest response time, but most of them had a low TCR. The monolayer MoS_2_ temperature sensor in this study showed a 100 times faster response than the other flexible temperature sensors. The response characteristics of the MoS_2_ temperature sensor were analyzed in detail by embedding a metal microheater in the sensor, as shown in Figure 7d. A negative voltage pulse was biased to the microheater to prevent the MoS_2_ sensor from accidentally turning on. As the pulse was biased to a heater, the current of the sensor increased immediately and decreased as the bias went to zero; the rising time was ~35 μs, and the falling time was ~29 μs, as shown in Figure 7e. In contrast, the sensor’s thermal response time was mainly limited to the PI substrate and Al_2_O_3_ layer. Therefore, thermal simulation was performed to estimate the intrinsic response time; the exponential signal was applied to the heater, and both the modeled and measured sensor time constants matched well (Figure 7f, left). A step function signal was then applied, and this temperature sensor could respond to temperature variations within a few μs (Figure 7f, right). Consequently, a 4 × 4 sensor array was fabricated for use in spatial mapping of the temperature (Figure 7g).

The bandgap of 2D TMDs was converted from an indirect bandgap to a direct bandgap to reduce the dimensionality from the bulk to a lower dimension. Therefore, outstanding electrical and optical performance can be achieved by controlling the bandgap [69]. Moreover, 2D TMDs, with their superior mechanical flexibility, facilitate the development of thin photodetectors on flexible materials such as PI and PET [70,71,72,73,74,75]. Among the 2D TMDs, MoS_2_ has excellent carrier mobility and high light conversion efficiency [76,77,78]. Their strong light-matter interaction was reflected in their high absorption coefficient of approximately 10^6^ cm^−1^, which was higher than that of standard semiconductors such as Si and GaAs [79]. In addition, molybdenum is naturally more abundant than other TMDs, making MoS_2_ transistors easier to manufacture [80,81].

**Figure 8:**
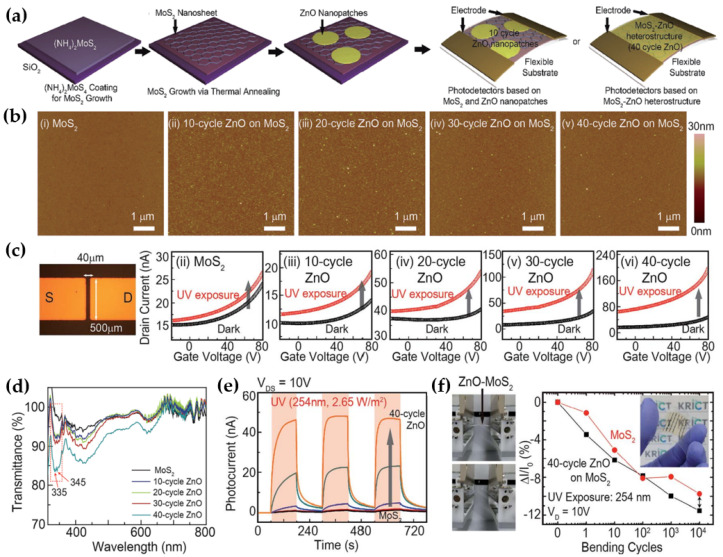
Flexible photodetector with MoS_2_ nanosheets and ZnO nanopatches. (**a**) Schematic diagram of the hybrid film synthesis. (**b**) AFM images of MoS_2_ nanosheets after the ALD cycle for ZnO deposition. (**c**) Optical image of the photodetector on the left. The optical performance comparison of the conventional MoS_2_ photodetector and MoS_2_–ZnO photodetector through the ALD cycle on the right. (**d**) Optical transmittance of the ZnO–MoS_2_ films on polyethylene terephthalate (PET) substrates for different ALD cycles. (**e**) Time-dependent photocurrent of MoS_2_–ZnO photodetector under UV light exposure at a V_d_ = 10 V for cycle: 10, 20, 30, and 40 cycles. (**f**) Bending test of the MoS_2_–ZnO hybrid photodetector in respect to the number of cyclic bending at a radius of 3 mm [82].

Kang et al. proposed a flexible photodetector based on MoS_2_ and ZnO hybrid films [82]. The MoS_2_ film was prepared via a hydrothermal process by spin-coating (NH_4_)_2_MoS_4_ dispersed in ethylene glycol on a SiO_2_ substrate and thermal annealing to obtain high-quality continuous MoS_2_ nanosheets. After MoS_2_ growth, ZnO nanopatches were then deposited on the MoS_2_ nanosheets via an ALD process. Finally, to produce photodetectors based on a MoS_2_-ZnO heterostructure, Cr (5 nm) and Au (60 nm) electrodes were fabricated on the MoS_2_-ZnO hybrid nanosheets, as shown in Figure 8a. Atomic force microscopy (AFM) was used to assess the surface roughness of the MoS_2_ nanosheets according to the number of ALD cycles for ZnO (Figure 8b). At 10 cycles, the roughness of the MoS_2_ nanosheets with ZnO nanopatches reached its maximum (0.57 nm) due to the large number of ZnO grains during the initial deposition. As the number of ZnO deposition cycles increased, the roughness decreased. The V_th_ of the MoS_2_ nanosheets moved towards a more positive voltage, and the photocurrent was significantly improved due to the excellent responsiveness of the ZnO film to UV light exposure, as shown in Figure 8c. The absorption peaks of 335 and 345 nm of hybrid film increased as the ZnO film thickness increased, as shown in Figure 8d. In addition, the time-dependent photocurrent responses in the UV region were investigated. Moreover, the photocurrent significantly increased as the number of ALD cycles of ZnO increased (Figure 8e). These results suggested that the ZnO thin film helps compensate for the numerous defects, such as defects and impurities in the relatively thin synthetic MoS_2_ film. As a result, the carrier transport characteristics of the heterostructure film were enhanced by decreasing the recombination rate of electron-hole pairs. The variation of photocurrent of the initial MoS_2_ and MoS_2_-ZnO for bending cycles was confirmed, as shown in Figure 8f. Both devices showed no dramatic difference during the bending cycles, indicating that the ZnO thin film does not degrade or undergo significant structural changes under mechanical stress.

**Figure 9:**
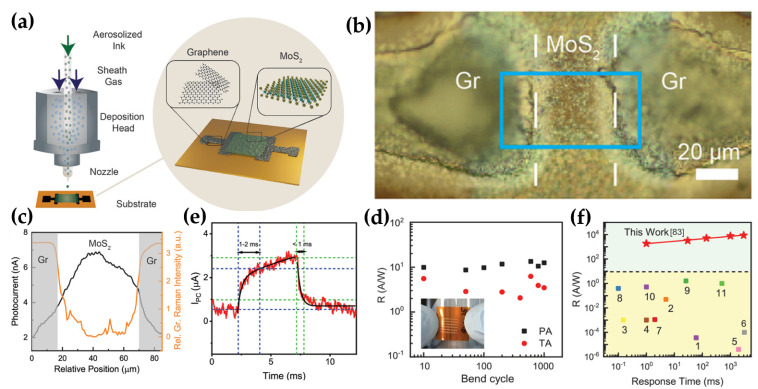
Flexible MoS_2_ nanosheet photodetectors. (**a**) Schematic diagram of aerosol jet printing (AJP) method and printed flexible photodetectors. (**b**) Optical microscopy (OM) image of the photonically annealed device. (**c**) Scanning photocurrent microscopy (SPCM) image of the horizontal line profile of photonically annealed device. (**d**) Mechanical stability of photonically annealed and thermally annealed devices at a bending radius of 12 mm. (**e**) Temporal photocurrent of photonically annealed photodetectors. (**f**) Comparison of the responsivity and response time with previous studies [83].

Kuo et al. proposed an ultrahigh–responsive photodetector with MoS_2_ nanosheets with graphene electrodes on a flexible PI substrate [83]. The MoS_2_ nanosheet and graphene electrodes were then deposited using aerosol jet printing (AJP) with MoS_2_ and graphene inks obtained through mega-sonic exfoliation, as shown in Figure 9a. Mild thermal annealing in an Ar atmosphere at 280 °C during 1 h or photonic annealing in ambient conditions at 2.8 kV for 1.36 ms was conducted to remove impurities and residues (polyvinylpyrrolidone and ethyl cellulose) from the AJP deposition process of MoS_2_ nanosheets. The photonically annealed photodetector structure of the top view was confirmed by optical microscopy (Figure 9b). Subsequently, the blue line area was mapped using scanning photocurrent microscopy (SPCM), indicating a higher photo response of the MoS_2_ region than the graphene region (Figure 9c). The flexibility and bending stability of the devices were evaluated through bending cycles of 10^3^ at a radius of 12 mm, and the photonically annealed device showed higher responsivity and stability, as shown in Figure 9d. The photonically annealed device exhibited higher optoelectronic performance because high intermixing between the MoS_2_ channel and graphene contacts was formed through photonic annealing using high-intensity pulsed light to heat locally and rapidly. The time-dependent photocurrent of the photonically annealed device at a wavelength of 515.6 nm with an intensity of 7 × 10^−5^ W/cm^2^ showed rise and fall time constants of the photocurrent of 1–2 ms and <1 ms, respectively (Figure 9e). These improved response time, compared to thermally annealed devices, were linked to the reduced density of trap states, cracks, and voids typically induced at high temperatures. Relative to previous studies, the flexible photodetector showed superior performance with rapid response time and higher responsivity, as shown in Figure 9f.

### 2.3. Displays

**Figure 10:**
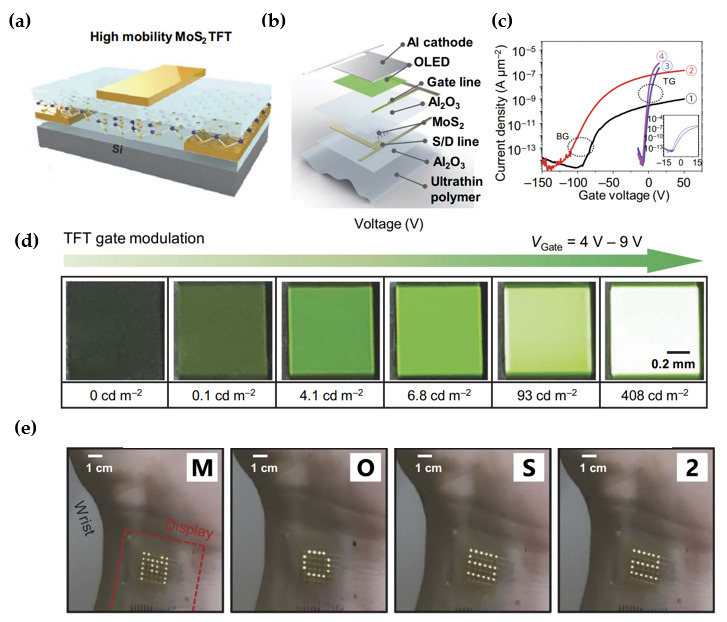
Flexible active-matrix OLED performance. (**a**) Schematic diagram of the MoS_2_ TFT with an Al_2_O_3_ layer. (**b**) Structures of each layer for ultrathin AM-OLED. (**c**) Transfer curves with respect to the top/bottom gate structure and the presence of dielectric Al_2_O_3_ in a MoS_2_ TFT. (①) Back gate MoS_2_ TFT on SiO_2_/Si, (②) Back gate MoS_2_ TFT on SiO_2_/Si with Al_2_O_3_ encapsulation, (③) Top gate MoS_2_ TFT on SiO_2_/Si, and (④) Top gate MoS_2_ TFT on Al_2_O_3_/SiO_2_/Si (④). (**d**) The luminance of an OLED following the intensity of V_g_ in the range from 4 V to 9 V. (**e**) Images of the operational dynamics of a fabricated AM-OLED display worn on a human wrist [84].

Various types of transistors were used as switching and driving devices in the backplane of the active-matrix display panels. The basic requirements of those transistors are rapid switching characteristics (high mobility) and high uniformity, and the performances of multiple transistors in the array exhibit equivalent levels. Choi et al. proposed the flexible active-matrix organic light-emitting diode (AM-OLED) backplane using MoS_2_ TFTs [84]. The electrical and mechanical properties of MoS_2_ are suitable for flexible AM-OLED and LED backplanes that require high carrier mobility and light transmittance [85,86,87]. The layer structure of a transparent and flexible AM-OLED display could be realized, as shown in Figure 10a. The metal-organic CVD-grown bilayer MoS_2_ channel layer was surrounded by Al_2_O_3_ layers with the top and bottom (Figure 10b) to achieve high mobility. The electrical characteristics were sequentially compared when forming Al_2_O_3_ on top of MoS_2_ and underneath MoS_2_ to demonstrate the effect of adopting Al_2_O_3_. When the Al_2_O_3_ layer was encapsulated on the bottom-gate structured MoS_2_ transistor on a SiO_2_/Si substrate, I_d_ increased dramatically compared to the TFT without Al_2_O_3_. This was attributed to the doping effect from the Al_2_O_3_ layer. In addition, the slight increase in I_d_ was confirmed when the structure was changed from a bottom-gate to a top-gate, and I_d_ was enhanced when adopting the Al_2_O_3_ layer under the MoS_2_ layer. The bottom Al_2_O_3_ layer affected the decrease in the trap charge density, resulting in an improvement of hysteresis and electrical characteristics. Interestingly, the 100 MoS_2_ transistors with an optimized structure exhibited relatively uniform performance, which is a requirement for transistors used in a pixel array (Figure 10c). The luminance of the OLED was increased from zero to 408 cd/m^2^ as V_g_ was increased from 4 V to 9 V, highlighting the well-operation of the TFT as a driving element in Figure 10d. Finally, the pixels with a 6 × 6 array were realized on an ultrathin PET substrate, and the OLED display, affixed to the wrist, responded immediately to the input signals, showing the selective operation with different letters in Figure 10e.

### 2.4. Memory Devices

**Figure 11:**
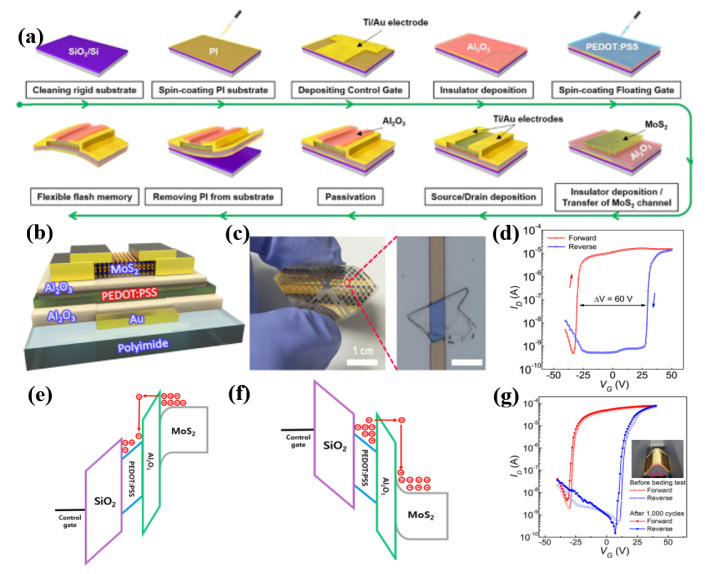
Flexible MoS_2_ flash memory and mechanical durability test. (**a**) Schematic diagram of the fabrication process of flexible MoS_2_ flash memory. (**b**) Cross-sectional structure of the device on a PI substrate. (**c**) Photograph and optical microscopy images of the device. (**d**) Transfer curves and memory window of flexible flash memory. Operation mechanism with energy band diagram for (**e**) programming and (**f**) erasing. (**g**) Transfer curves of 1000 bending cycles with a bending radius of 5 mm [100].

Alternative applications of 2D MoS_2_ include flexible memory devices with characteristics such as gate-tunable meristic behavior [88,89,90,91] and optoelectronic properties [92,93,94]. Novel materials, structures, and fabrication processes have been investigated to develop flexible memory devices based on MoS_2_ transistors [37,95,96,97,98,99]. Hong et al. proposed the flexible MoS_2_ flash memory devices and their fabrication using a multilayered MoS_2_ channel, poly (3,4-ethylenedioxythinophene):poly (styrene sulfonate) (PEPOT:PSS) floating gate layer, and PI substrate [100]. Each layer of flexible MoS_2_ flash memory was fabricated on the PI spin-coated rigid Si/SiO_2_ substrate, and the flexible device was then obtained by desquamating from a Si/SiO_2_ substrate in Figure 11a. Figure 11b,c present cross-section and top view images of flexible MoS_2_ flash memory, respectively; 80 nm- and 100 nm-thick Al_2_O_3_ films on PEDOT: PSS were used as a tunneling dielectric layer (TDL) and blocking dielectric layer (BDL), respectively. Negligible hysteresis with almost no memory window was observed when only the MoS_2_ transistor was used as a flash memory. On the other hand, by adopting a PEDOT:PSS as a floating gate, a large hysteresis, with a memory window of 54.6 V, was observed in the flash memory on the rigid substrate in Figure 11d. Similarly, a large memory window (60 V) was also observed when the flash memory was fabricated on the PI substrate (Figure 11d). The dramatic increase in the memory windows, storing data as memory, was possible because of the charge-trapping role of PEDOT:PSS. When biasing at a 60 V gate voltage, V_program_, electrons pass through the TDL and are trapped in the PEDOT:PSS layer (Figure 11e). By contrast, biasing at a −60 V gate voltage, V_erase_, trapped electrons in PEDOT:PSS pass through the TDL and transported them to the MoS_2_ channel layer (Figure 11f). Furthermore, the flash memory could be applied to photoinduced memory, which can store information on the light intensity because the MoS_2_ and PEDOT:PSS layers can generate photoactivated carriers under the illumination wavelength of 405 nm. The characteristics of 1000 bending cycles at a bending radius of 5 mm barely changed, meaning high mechanical durability of flexible MoS_2_ memory in Figure 11g.

### 2.5. Logic Circuits

**Figure 12:**
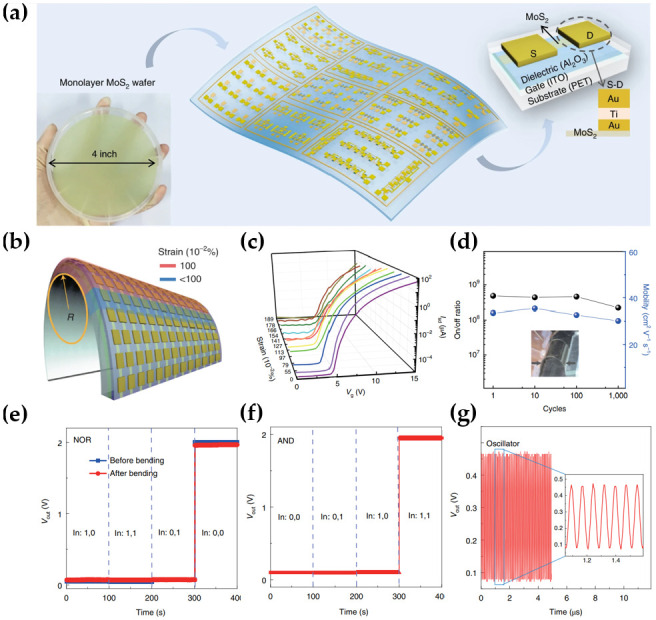
(**a**) Schematic diagram of a flexible transistor array with integrated multi-stage circuits, including various logic gates and ring oscillators. The lower left is a single MoS_2_ film enlarged to four inches using epitaxy technology, and the upper right is the structure of a flexible MoS_2_ FET. (**b**) Schematic diagram of the transistor array strain under bending conditions. (**c**) I–V characteristics of the flexible MoS_2_ FETs at variant strain magnitudes. (**d**) I_on_/I_off_ ratio and charge-carrier mobility of the MoS_2_ TFT under 1000 cycles of bending stress. (**e**) Output characteristics of the NOR gate with bending stress. (**f**) Output characteristics of AND gate consisting of inverter and NAND. (**g**) Waveform output of a five-stage ring oscillator [34].

Based on the high compatibility and flexibility of MoS_2_, the material has been used for the fabrication of flexible integrated circuits as a part of logic circuits [47,101,102,103,104,105]. Li et al. proposed a large-scale flexible monolayer MoS_2_ transistor array with high performance [34]. The CVD system was reconfigured to achieve a four-inch wafer-scale MoS_2_ film with large grain sizes. This array contained integrated multi-stage circuits (Figure 12a). Among them, the transistors were composed of an indium thin oxide (ITO) gate electrode, an Al_2_O_3_ gate dielectric, a MoS_2_ monolayer, and Au/Ti/Au source and drain electrodes. Generally, Au/Ti electrodes are used widely as the source and drain electrodes for MoS_2_ to improve the adhesion between the electrodes and MoS_2_. On the other hand, Au/Ti/Au electrodes were used to lower the contact resistance, forming ohmic contact with the MoS_2_ and Au while maintaining good adhesion by forming a Ti-mixed Au layer. Although these FETs were realized on a large scale, the distribution in device-to-device transfer curves was relatively uniform in the 10 × 10 transistor array, showing an overall yield of 97% with small hysteresis. As shown in Figure 12b, they tested the convex bending of the flexible transistors, performing a 2% tensile strain at the highest bending strain. Even at a strain of up to ~1%, MoS_2_ FETs exhibited relatively consistent electrical performance (Figure 12c). In addition, the I_on_/I_off_ ratio and mobility of MoS_2_ FETs in this array remained stable after 1000 bending cycles, confirming the high mechanical stability, as shown in Figure 12d. The input voltage was set to 0 and 1 when V_dd_ was 2 V to make the basic logic function more complex. Stable performance was observed even after bending, as shown in Figure 12e. This performance was the same for the SRAM devices. Regarding the output characteristics of the AND gate, which is another gate composed of an inverter and NAND, 0 V and 2 V were set as before to correct the input voltage over time (Figure 12f). When V_dd_ = 15 V, the rapid stage delay (τ) with an oscillation frequency of 13.12 MHz was 7.6 ns, which corresponds to a high-tech flexible ring oscillator, as shown in Figure 12g.

### 2.6. Neuromorphic Devices

**Figure 13:**
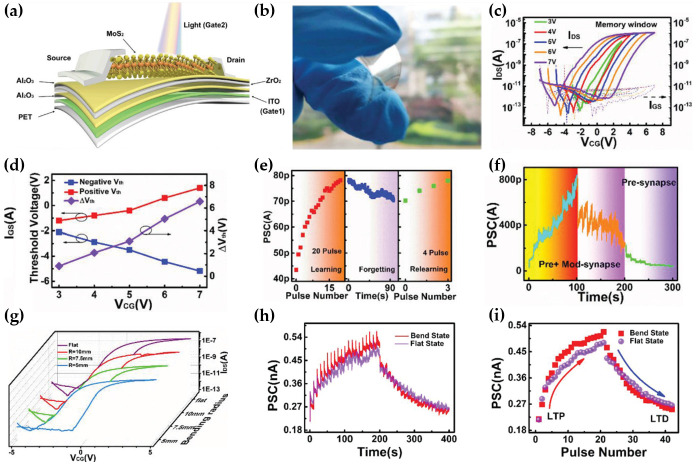
Flexible heterosynapse device based on a MoS_2_ transistor. (**a**) Schematic structure of the MoS_2_ heterosynapse device. (**b**) The photograph of a flexible artificial neuromorphic device. (**c**) Transfer characteristics of MoS_2_ memory. (**d**) Memory window with large hysteresis. (**e**) Learning and forgetting emulation by light-induced modulatory synapses. (**f**) Electrical and optical pulses induced LTP and LTD. (**g**) I–V characteristics at different bending radii (flat, 10, 7.5, and 5 mm). (**h**) EPSC and IPSC stimulated with electrical pulses (amplitude = −4 V/5 V, number of pulses = 20, pulse width = 100 ns) per 10 s in flat and banding states (R = 10 mm). (**i**) Post-synaptic current variation depending on the number of pre-synaptic pulses in bent states (radius = 10 mm) and flat states [35].

The von Neumann structure, a traditional memory system, has a limitation in that it consumes a large amount of energy when processing large amounts of computing data. Neuromorphic computing technology is emerging as a next-generation memory system that can overcome the von Neumann bottleneck through low-power consumption, high-speed, and high-capacity data processing [106,107,108]. Wang et al. proposed a MoS_2_-based heterosynaptic module capable of low-power and ultrafast operation [35]. In most studies, neuromorphic techniques were implemented with simple plasticity, such as pre- and post-synaptic learning. On the other hand, they revealed a multi-terminal network with heterosynapses, including pre-, post-, and modulatory synapses. Figure 13a,b present a heterosynaptic structure composed of a PET substrate, ITO serving as a gate electrode by an electrical signal, an Al_2_O_3_/ZrO_2_/Al_2_O_3_ gate dielectric, an exfoliated MoS_2_ channel, of Ti/Pt source and drain electrodes. The transfer characteristics of the electrical mode in Figure 13c exhibited a distinct clockwise hysteresis window. The memory window, ΔV_th_, gradually became larger as the maximum intensity of the control gate voltage (|*V*_CG,max_|) increased, as shown in Figure 13d. Such behaviors were suitable for use as a non-volatile memory, and the operation mechanism is as follows. When the gate was applied to a positive voltage, electrons in the MoS_2_ layer migrated through the 5 nm thick tunneling layer (Al_2_O_3_) and accumulated in the charge-trapping layer (ZrO_2_). The accumulated electrons screen the gate electric field, resulting in an increase in V_th_. In contrast, when the gate was applied to a negative voltage, the electrons accumulated in a charge-trapping layer tunnel migrated back to the MoS_2_ layer. The decreased quantity of electrons in a charge-trapping layer weakens the screening effect against the gate electric field, resulting in a decrease in V_th_. Using this operation mechanism, inhibitory post-synaptic current (IPSC) and excitatory photo-synaptic current (EPSC) were measured by electrical pulses to reveal the potentiation and depression behaviors, respectively. Furthermore, the advanced learning experience behavior was emulated by adding optical modulation with light pulses. The same process as the first learning step was repeated following the initial 20 light pulses and waiting for the PSC to collapse spontaneously (Figure 13e). On the other hand, the same value of PSC could be obtained with only four light pulses, meaning that learning time decreased as learning was repeated, like a human brain. A higher order of long-term potentiation (LTP) was emulated using electrical and optical pulses simultaneously, but additional electrical pulses were required to return to their initial state because of weakened depression behavior, as shown in Figure 13f. Finally, the I-V characteristics under the bending state were measured to reveal the flexibility of MoS_2_-based artificial synapses. The ΔV_th_ of 2.4 V, 2.4 V, and 3 V was observed when the bending radius was 10 mm, 7.5 mm, and 5 mm, respectively, indicating negligible degradation (Figure 13g). Moreover, they successfully emulated the LTP and long-term depression (LTD) behavior under bending states, and the operations were reliable without severe degradation, as shown in Figure 13h,i.

## 3. Conclusions

This paper introduced a wide range of applications in recent research over the past decade on flexible electronics based on MoS_2,_ which is representative 2D TMD material. In particular, the 2D MoS_2_ exhibits high sensitivity, selectivity, flexibility, and easy formability with various methods. Therefore, the 2D MoS_2_-based transistors, which serve as the basis for wearable electronics, have been investigated, even though the other 2D TMD materials-based transistors showed higher performance. Nevertheless, flexible MoS_2_-based transistors still exhibit relatively poor performance because of the increased difficulty in fabricating stable devices originating from the large changes in the existing processes and materials. Novel structures, materials, and fabrication processes should be further developed to achieve similar or superior performance compared to conventional silicon-based devices because flexible transistors use flexible polymer substrates, such as PI and PET, instead of conventional rigid substrates. This paper introduced distinguishing process sequences, materials, and structures, which are used for improving the mechanical stability and electrical characteristics of flexible MoS_2_ transistors. Furthermore, various applications using their optimized methods were discussed: multifunctional sensors on contact lenses, ultrasound detectors, active-matrix pressure sensors, rapid-response temperature sensors, photodetectors, active-matrix OLEDs, flash memory devices, logic circuits, and neuromorphic devices (Table 1). Wearable electronics based on MoS_2_ have high potential and will offer an extensive range of applications in more diverse fields.

## 4. Future Directions

Research on wearable electronics is emerging as a core technology in various fields, such as robotics, medical applications, entertainment, security, and display fields. Wearable electronics require high-performance flexible transistors. Therefore, 2D TMDs, including MoS_2_-based transistors, are promising candidates. Although MoS_2_, a representative TMD, has many advantages, such as being easy to fabricate transistors on a flexible substrate, tunable bandgap, and excellent carrier mobility, there is an additional need for the development of other TMD materials exhibiting unique advantages. Research has expanded beyond MoS_2_ to various TMDs, including molybdenum diselenide (MoSe_2_), tungsten disulfide (WS_2_), and tungsten diselenide (WSe_2_), each with unique properties and potential applications. Researchers are recommended to investigate various processes, such as post-treatment, doping, and supportive layer, for each TMD material-based transistor.

The major issue as to whether 2D TMDs can be commercialized has been ongoing since the first discovery of 2D TMDs. On the other hand, significant challenges remain before 2D TMD-based transistors can be commercialized. First, one of the major challenges is forming 2D TMDs uniformly over a large area. For commercialization, it is crucial to demonstrate high throughput and uniform characteristics. Therefore, focusing on uniformly depositing or growing 2D TMDs on the large wafer is underway and is considered the leading research with high impact in this field. The next step is to develop technology that achieves the high uniformity of a large number of 2D TMD transistors within a wafer, comparable to conventional silicon-based technology. Another challenge limiting commercialization is the stability of the electrical characteristics. When using electronics, the performance of transistors should remain stable over an extended period to prevent malfunctions. Nevertheless, 2D TMD transistors generally exhibit poor electrical stability because the extremely thin TMD layers are vulnerable to external environments and large electric fields. In this respect, enhancing the intrinsic reliability of TMD materials and developing encapsulation techniques should be investigated.

Furthermore, several aspects need to be verified when applying 2D TMDs for wearable electronics. The electronics are worn directly on human skin. Therefore, in the advanced form of wearable electronics, the 2D TMD transistors need to exhibit stretchable properties like the skin, and cytotoxicity must be considered. Stretching imposes more severe mechanical stress than bending. Hence, the platform structure for 2D TMD transistors needs to be optimized to withstand this stress. Research on applying MoS_2_ transistors to essential parts of the body is also underway, and they have not yet shown fatal toxicity to the human body. For safety, biocompatibility, including cytotoxicity against other TMD-based transistors, must be determined before they can be implemented as complete wearable electronics. If technologies address these issues are developed, the long-held skepticism towards the commercialization and wide-ranging application of 2D TMD-based transistors may transform into a reality.

## Figures and Tables

**Table 1:** Comparison of flexible and wearable electronics based on MoS_2_ transistors.

Application		Fabrication	Layers of MoS_2_	Device Structure	Types of Substrates	I_on_/I_off_	µ_FE_ (cm^2^ V^−1 ^s^−1^)	Mechanical Stability		Ref.
								Strain	Bending Radius	
Transistor		CVD	Monolayer	Top gate	Polyimide (PI)	>10^6^ (V_ds_ = 1.4 V)	8.1 (**µ_FE,ext_**)	Tensile strain of <0.063%	R = 4 mm	[49]
		Exfoliation	Multilayer	Top gate	PI/Polyethylene terephthalate (PET)	5 × 10^5^ (V_ds_ = 1 V)	141.3	Total strain of <0.2%	R = 5–10 mm	[50]
Sensor	Photo/glucose/temperature	Gold-mediated exfoliation	Monolayer	Top gate	Polydimethylsiloxane (PDMS)	10^7^ (V_ds_ = −5 V)	9.18	Tensile strain of <2.0%	R > 4 mm	[32]
	Sonic detector	Exfoliation	Multilayer	Top gate	PI/PET	10^5^ (V_ds_ = 1.4 V)	18.12	Total strain of <0.1%	R = 5 mm	[65]
	Pressure	CVD	Monolayer	Top gate	PDMS	>10^6^ (V_ds_ = 1 V)	≈23	Remain stable during 2 × 10^3^ bending cycles	-	[33]
	Temperature	CVD	Monolayer	Top gate	PI	≈10^8^ (V_ds_ = 1 V)	≈20	Remain stable during 10^3^ bending cycles	R = 4 mm	[66]
	Photo	Spin coating	Multilayer	Top gate	PET	-	-	Total strain of <3%	R = 3 mm	[82]
		Megasonic exfoliation (MSE)	Mono- and bilayer	Bottom gate	PI	-	0.7	Remain stable during 10^3^ bending cycles	R = 12 mm	[83]
Display		CVD	Bilayer	Bottom gate	PET	≈10^8^ (V_ds_ = 5 V)	18.1	Total strain of <3%	R = 0.7 mm	[84]
Memory device		Exfoliation	Multilayer	Top gate	PI	10^7^ (V_ds_ = 1 V)	-	Remain stable during 10^3^ bending cycles	R = 5 mm	[100]
Login circuit		CVD	Monolayer	Top gate	PET	10^10^ (V_ds_ = 1 V)	≈55	Tensile strain of <1%	-	[34]
Neuromorphic device		Exfoliation	Multilayer	Top gate	PET	>10^6^ (V_ds_ = 0.5 V)	-	Remain stable during 10^3^ bending cycles	R = 5 mm	[35]
